# Should we floss our nose? A case report of a tooth in the nasal cavity

**DOI:** 10.1016/j.ijscr.2021.105664

**Published:** 2021-03-01

**Authors:** Ahmed Khalid Ahmed, Hasan Alansari, Abdulrahman Almannai, Hesam Yusuf Ali

**Affiliations:** King Hamad University Hospital, Bahrain

**Keywords:** Rhinology, Pediatrics, Endoscopy

## Abstract

**Introduction and importance:**

Supernumerary teeth are an anomaly characterized by the existence of an additional tooth that may grow in the dental arch region, it a rare occurrence and may be confused with other differential diagnosis, it is important treat such case as they can cause considerable morbidity to the patient.

**Case presentation:**

We report a case of a 16 years old female, not known case of any medical condition, who presented to the Ear, Nose and Throat clinic complaining of nasal obstruction and rhinorrhea. Physical examination and CT findings revealed a right upper central incisor tooth located in anterior aspect of right inferior meatus. The patient underwent endoscopic teeth extraction under general anesthesia.

**Conclusion:**

Supernumerary tooth is a rare condition, often causes significant distress to patients and often misdiagnosed in a clinical setting. A thorough history physical examination including the anterior rhinoscopy and nasal endoscopy needs to be done for all patients. Further radiological investigations such as CT scan of the paranasal can aid and help confirm the diagnosis. The most common treatment is usually trans nasal endoscopic removal of the tooth.

## Highlights

• Supernumerary teeth is an anomaly characterized by the existence of an additional tooth that may grow in the dental arch region, it a rare occurrence and may be confused with other differential diagnosis.
• 16 years old female, not known case of any medical condition, who presented to the Ear, Nose and Throat clinic complaining of nasal obstruction and rhinorrhea.
• Nasal endoscopy of the right nostril showed white solid nasal mass arising from the floor right nasal cavity covered in excess secretions and discharge.
• CT paranasal sinus showed enveloped focal soft tissue density, insinuated between the medial surface of the right inferior turbinate and bony nasal septum.
• Decision was then taken to remove the tooth endoscopically in the operating theatre while was under general anaesthesia.
• The pathology reported it as a fully formed tooth.

## 1 Introduction

A supernumerary tooth is defined as an additional tooth that may grow anywhere along the dental arch region, such teeth have been known to grow in various areas such as the mandible, maxilla, palate [1]. It is reported in literature to have a prevalence 0.1–3.5% [2]. To have it grow along the nasal floor is a rare occurrence and can often be confused with other differentials in a clinical setting. The symptoms are often nonspecific and might cause distress and discomfort for a patient. We report a case of fully formed supernumerary central incisor tooth in the right nasal cavity of a 16-year-old female patient.

## 2 The case

A 16-year-old female presented to the Ear, Nose and Throat clinic complaining of a long standing history of nasal obstruction and rhinorrhea mostly on the right side. She had no history of facial pain, anosmia, epistaxis or headache. The patient was medically free and had no previous diagnosis of any sort of syndrome or had previous ear, nose and throat procedures. Patient had no relevant family or social history.

On anterior rhinoscopy, the patient had a right side deviated nasal septum along with mucopurulent discharge. The nasal endoscopy of the right nostril showed white solid nasal mass arising from the floor right nasal cavity covered in excess secretions and discharge. The left side was normal and the nasopharynx was clear as well, examination of throat and neck revealed no abnormalities.

**Image 1:**
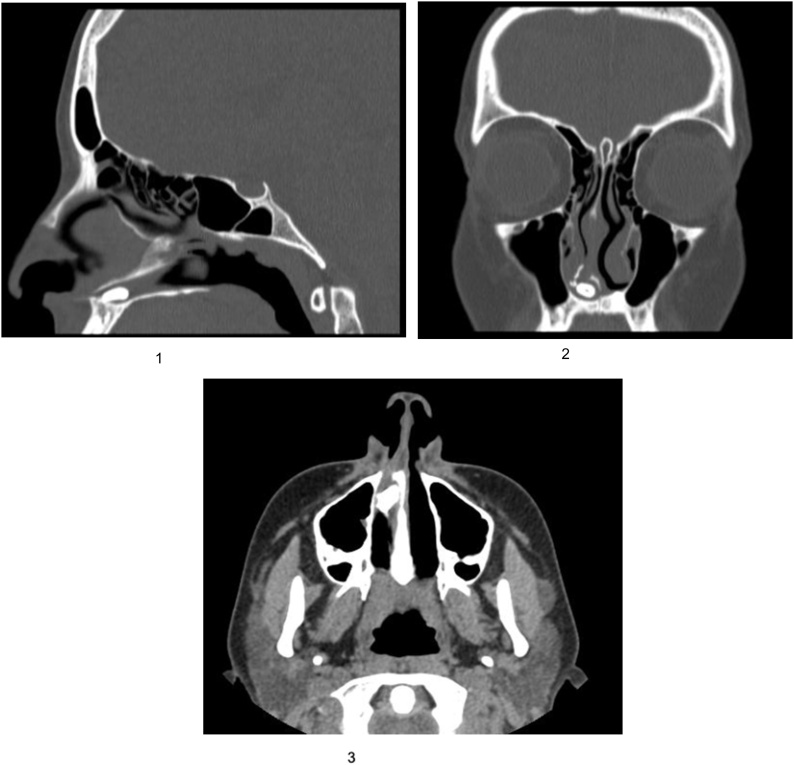
(1) Sagittal View CT-scan. (2) Coronal View CT-scan. (3) Axial View CT-Scan.

The patient then underwent a non-contrast CT paranasal sinus (Image 1 (1), (2), (3)) and it was reported as an enveloped focal soft tissue density, insinuated between the medial surface of the right inferior turbinate and bony nasal septum.

**Image 2:**
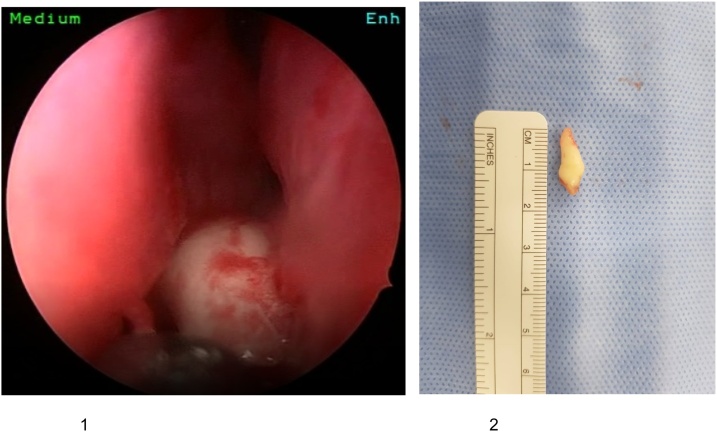
(1) Endoscopic view of tooth. (2) Endoscopic extracted tooth.

The decision was then taken to remove the tooth endoscopically in the operating theatre by the consultant while she was under general anesthesia. Intraoperatively the patient was found to have excess muco-purulent discharge around the area of the tooth and the mucosa was friable and indurated. The tooth was found in the area as it was described in the CT scan (Image 2 (1)) and was extracted endoscopically in its full form (Image 2 (2)).

**Image 3:**
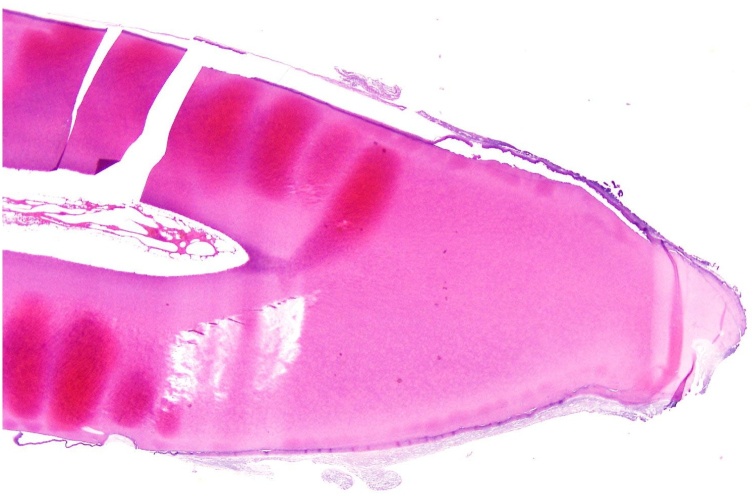
Histopathology slide of the tooth.

The pathology reported it as a fully formed tooth. Morphologically, it is consistent with an unerupted right central incisor structure. The full length of the tooth all the way to the crown was well-displayed. The tooth was also described as quite viable including the pulp cavity (Image 3).

The patient was discharged from the hospital with no complications. She was seen again in the ENT clinic 1 week later. The mucosa of the right side of the nose healed well and there were no signs of recurrence or complications.

## 3 Discussion

As mentioned previously, supernumerary tooth growth in the nasal cavity is a rare occurrence, it tends to be found in males more than females. It can cause a wide range of symptoms including but not limited to pain in the nasal area, nasal discharge nasal obstruction, rhinorrhea, headache, and epistaxis [3,4], the exact cause of it remains to be clear but it has been theorized to be caused by developmental abnormalities, facial traumas or even due to overcrowding of the teeth causing an abnormal eruption [5]. Conditions such as gardeners and cleidocranial dystosis are also known to be associated with development of a supernumerary tooth [6].

Examination of such patient would usually reveal a unilateral nasal mass in the nasal cavity, the tooth is usually covered by mucosa, nasal secretions and granulation tissues, the differential diagnosis is wide in such patients should include, foreign body, rhinolith, bony sequestration and even neoplasm, so it is important to that these patients are carefully examine [3,5,7].

The use of radiological investigations can aid us in diagnosis are the ophthantogram and CT paranasal sinuses, such investigation could actually show a radiopaque mass and in some cases can even confirm the diagnosis which was the case with our patient [2,3].

Prompt treatment is important because the supernumerary tooth may cause considerable pain and discomfort to the patient, it presence in the nasal cavity will lead to inflammation which may possible cause further nasal mucosa injury, epistaxis, sinusitis, septal abscesses and even oro-nasal fistulas [[8], [9], [10]].

Treatment is extraction of the tooth, it is usually done by endoscopic view trans-nasal, care must be take to remove the tooth completely and carefully as it wise to avoid further mucosal injury, this could be done in the clinic under local anesthesia if the patient is co-operative or in the operating theatre under general anesthesia, other methods of tooth extraction describe in the literature would be the trans-palatal approach but it is not favored due to the fact that the trans nasal approach offer better visualization and causes much less post-operative morbidity [3,11,12]. Our case report was written as per the SCARE 2020 guidelines [13].

## 4 Conclusion

Supernumerary tooth is a rare condition, often causes significant distress to patients and often misdiagnosed in a clinical setting. A thorough history physical examination including the anterior rhinoscopy and nasal endoscopy needs to be done for all patients. Further radiological investigations such as CT scan of the paranasal can aid and help confirm the diagnosis. The most common treatment is usually trans nasal endoscopic removal of the tooth.

## 5 Learning objectives

• Intranasal supernumerary tooth is a rare finding which is only present in 0.1% to 3.5% of the population
• The symptoms are unspecific and commonly misdiagnosed due to the wide list of differential diagnosis
• The use of radiology such as CT and paranormic X-ray is beneficial in diagnosis of supernumerary tooth
• Prompt treatment is important due to further complications that can caused by it.
• Trans-nasal endoscopic approach is the most common surgical approach to treat supernumerary tooth cases

## Declaration of Competing Interest

The authors state that they have no conflicts of interest for this report.

## Funding

This research did not receive any specific grant from funding agencies in the public, commercial, or not-for-profit sectors.

## Ethical approval

This case report is exempt from ethical approval at our institution.

## Consent

Written informed consent was obtained from the patient for publication of this case report and accompanying images. A copy of the written consent is available for review by the Editor-in-Chief of this journal on request.

## Registration of research studies

Not Applicable.

## Guarantor

Professor Hesam Yusuf Ali.

## Provenance and peer review

Not commissioned, externally peer-reviewed.

## CRediT authorship contribution statement

**Ahmed Khalid Ahmed:** Conceptualization, Data curation, Formal analysis, Methodology, Project administration, Resources. **Hasan Alansari:** Conceptualization, Data curation, Formal analysis, Funding acquisition, Investigation, Methodology, Project administration, Supervision, Validation, Visualization, Writing - original draft, Writing - review & editing. **Abdulrahman Almannai:** Conceptualization, Data curation, Formal analysis, Validation, Visualization, Writing - original draft, Writing - review & editing. **Hesam Yusuf Ali:** Supervision, Validation, Visualization, Writing - original draft, Writing - review & editing.
